# Epidemiology and outcomes of traumatic chest injuries in children: a nationwide study in the Netherlands

**DOI:** 10.1007/s00431-023-04828-1

**Published:** 2023-02-18

**Authors:** Arthur A. R. Sweet, Ivar G. J. de Bruin, Jesse Peek, Frank F. A. IJpma, Mark C. P. M. van Baal, Luke P. H. Leenen, Roderick M. Houwert

**Affiliations:** 1 Department of Surgery, University Medical Center Utrecht, Utrecht, The Netherlands; 2 Department of Surgery, University Medical Center Groningen, Groningen, The Netherlands

**Keywords:** Pediatrics, Thoracic trauma, Epidemiology, Injury patterns, Outcomes

## Abstract

Thoracic injuries are infrequent among children, but still represent one of the leading causes of pediatric mortality. Studies on pediatric chest trauma are dated, and little is known of outcomes in different age categories. This study aims to provide an overview of the incidence, injury patterns, and in-hospital outcomes of children with chest injuries. A nationwide retrospective cohort study was performed on children with chest injuries, using data from the Dutch Trauma Registry. All patients admitted to a Dutch hospital between January 2015 and December 2019, with an abbreviated injury scale score of the thorax between 2 and 6, or at least one rib fracture, were included. Incidence rates of chest injuries were calculated with demographic data from the Dutch Population Register. Injury patterns and in-hospital outcomes were assessed in children in four different age groups. A total of 66,751 children were admitted to a hospital in the Netherlands after a trauma between January 2015 and December 2019, of whom 733 (1.1%) sustained chest injuries accounting for an incidence rate of 4.9 per 100,000 person-years. The median age was 10.9 (interquartile range (IQR) 5.7–14.2) years and 62.6% were male. In a quarter of all children, the mechanisms were not further specified or unknown. Most prevalent injuries were lung contusions (40.5%) and rib fractures (27.6%). The median hospital length of stay was 3 (IQR 2–8) days, with 43.4% being admitted to the intensive care unit. The 30-day mortality rate was 6.8%.

*Conclusion*: Pediatric chest trauma still results in substantial adverse outcomes, such as disability and mortality. Lung contusions may be inflicted without fracturing the ribs. This contrasting injury pattern compared to adults underlines the importance of evaluating children with chest injuries with additional caution.

**Table:** 

**What is Known:** *• Chest injuries are rare among children, but represent one of the leading causes of pediatric mortality.* *• Children show distinct injury patterns in which pulmonary contusions are more prevalent than rib fractures.*	
**What is New:** *• The proportion of chest injuries among pediatric trauma patients is currently lower than reported in previous literature, but still leads to substantial adverse outcomes, such as disabilities and death.* *• The incidence of rib fractures gradually increases with age and in particular around puberty when ossification of the ribs becomes completed. The incidence of rib fractures among infants is remarkably high, which is strongly suggestive for nonaccidental trauma.*

**What is Known:**

*• Chest injuries are rare among children, but represent one of the leading causes of pediatric mortality.*

*• Children show distinct injury patterns in which pulmonary contusions are more prevalent than rib fractures.*

**What is New:**

*• The proportion of chest injuries among pediatric trauma patients is currently lower than reported in previous literature, but still leads to substantial adverse outcomes, such as disabilities and death.*

*• The incidence of rib fractures gradually increases with age and in particular around puberty when ossification of the ribs becomes completed. The incidence of rib fractures among infants is remarkably high, which is strongly suggestive for nonaccidental trauma.*

## Introduction

Thoracic injuries are infrequent among children, with an incidence ranging between 4 and 10% of all pediatric trauma patients [1–4]. Although uncommon, chest injuries represent one of the leading causes of death in pediatric trauma, especially in patients with concomitant abdominal or head injuries [1]. A more recent study, on the injury patterns of moderately to severely injured pediatric patients, showed that all children who sustained an injury to the chest had one or more concomitant injuries in other body regions [5]. The mortality rate among these patients was 13.9%. In pediatric polytrauma patients, chest injuries are seen more commonly, with almost two-thirds of these patients sustaining an injury to the chest [6].

Previous research has shown several differences in trauma mechanisms and injury patterns between pediatric and adult chest trauma patients [7]. Reasonably, children are more frequently injured as a result of pedestrian collisions compared to adults; moreover, injuries in children may also be caused by nonaccidental traumas (i.e., child abuse) [7]. An extensive national study in the United States reported that 3% of all children admitted after a trauma had injuries caused by nonaccidental trauma, with a mortality rate of 9%, mostly caused by head or chest injuries [8]. Other studies have shown that nonaccidental trauma was the most common cause of injuries among infants and toddlers, in which rib fractures had the highest probability of being caused by physical abuse of all injuries [9, 10].

In contrast to adults, ribs of young children are not completely ossified yet, resulting in an increased compliance of the chest wall [11, 12]. This causes an injury pattern in which pulmonary contusions are the most prevalent injuries in pediatric chest trauma, followed by the relatively uncommon rib fractures and pneumothorax [4, 7, 13–15]. This distinct injury pattern in which lung contusions are more prevalent than rib fractures underlines the importance of distinguishing between adults and children when evaluating chest injuries.

Studies on epidemiology of children with chest injuries are dated, and little is known about outcomes in different child ages. Therefore, this study aims to provide an overview of the current incidence, injury patterns, and in-hospital outcomes of children with chest injuries.

## Methods

The Medical Ethical Review Board of the University Medical Center Utrecht approved this study and granted a waiver of consent. This article was written in adherence to the STROBE (Strengthening the Reporting of Observational Studies in Epidemiology) statement [16].

### Patient selection

The present study describes a nationwide retrospective cohort study of children with chest injuries. Data were derived from the Dutch Trauma Registry (DTR), which covers approximately 99% of all hospitals in the Netherlands and prospectively collects data on all trauma patients acutely admitted to the hospital. All patients who were admitted to the hospital with injuries to the chest between January 2015 and December 2019 were identified using abbreviated injury scale (AIS) codes for chest injuries [17].

Patients below 16 years of age who sustained moderate (i.e., AIS thorax of 2) to most severe (i.e., AIS thorax of 6) chest injuries were included. To be able to provide a complete overview of the number of children with one or more rib fractures, we also added the AIS codes for one rib fracture to the search, as this injury is actually classified into an AIS thorax of 1. The included patients were stratified in four groups: 0–3 years, 4–7 years, 8–11, and 12–15 years.

### Incidence rates

The incidence rate of pediatric hospital admissions with moderate to severe thoracic injuries based on all children living in the Netherlands during our study period was calculated using national demographic data from the Dutch Population Register from the Central Bureau of Statistics [18]. Age-specific incidence rates of children admitted to the hospital after a trauma, with any chest injury, with a moderate to severe chest injury, and with one or more rib fractures were calculated using data from the DTR.

### Explanatory variables

The following baseline variables were obtained from the DTR: age at trauma, sex, American Society of Anesthesiologists (ASA) score, and mechanism of injury. Prehospital data were mode of transport (i.e., ambulance, own transport, or trauma helicopter), Glasgow coma scale (GCS), involvement of the mobile medical team (MMT), intubation on-scene, need for emergency intervention, and circulatory arrest before admission. The MMT consists of a trauma surgeon or anesthesiologist and a trained nurse to provide acute care at the scene of the accident. In the Dutch situation, a patient is rarely transported by helicopter due to short distances. Prehospital data on systolic blood pressure and respiratory rate were available, but these were not described as data were missing in almost half of the patients of certain age categories and the exact moment of measurement was unclear. Injury-related characteristics include number of rib fractures, presence of a flail chest, fractures of the sternum, clavicle and scapula, intrathoracic injuries (i.e., major pneumothorax (> 50% collapse of lung), major hemothorax (> 1000 cc blood loss on one side), major hemopneumothorax (> 1000 cc blood loss on one side), and lung contusion), tracheal injury, thoracic aorta dissection or rupture, concomitant abdominal and spinal injuries, revised trauma score (RTS), injury severity score (ISS) [19], number of polytrauma patients, and AIS scores for all body regions. The revised trauma score is a widely used 13-point scoring tool to determine the initial trauma severity based on the GCS, systolic blood pressure, and respiratory rate. A lower score reflects a higher severity of injury.

### Outcome measures

In-hospital outcome variables were time between trauma and arrival at the emergency department (ED), ED length of stay (ED-LOS), time between trauma and first intervention in case there was an emergency intervention, total number of patients admitted to a level 1 trauma center (i.e., a trauma center equipped to treat the most severely injured patients) and number of patients admitted to a level 1 trauma center in case of polytrauma, hospital length of stay (HLOS), admission to intensive care (ICU), ICU length of stay (ILOS), number of patients requiring mechanical ventilation, duration of mechanical ventilation (DMV), destination after discharge, Glasgow outcome scale (GOS) score at the time of hospital discharge, and in-hospital mortality. Additionally, the 30-day mortality was described as well.

### Statistical analysis

Data were analyzed using descriptive statistics and presented as frequencies with percentages for categorical data, means with standard deviations (SD) for parametric continuous variables, and medians with interquartile ranges (IQR) for nonparametric continuous variables. To assess whether variables followed a normal distribution, the Shapiro–Wilk test and Quantile–Quantile plots were performed. Incidence rates based on the complete Dutch child population were described per 100,000 person-years. Statistical analyses were performed using SPSS statistical software (SPSS 23.0; IBM Inc., Armonk, NY, USA).

## Results

### Incidence

During the study period, the Netherlands had approximately 17 million inhabitants of which 694,743 children aged 0–3 years, 731,587 children aged 4–7 years, 756,659 children aged 8–11 years, and 804,554 children aged 12–15 years. The incidence rate of children admitted to the hospital with moderate to severe chest injuries based on the complete Dutch child population was 4.9 per 100,000 person-years.

Over a five-year period, 66,751 children under 16 years of age were admitted to a hospital in the Netherlands between January 2015 and December 2019 (Table 1). Chest injuries were seen in 1602 (2.4%) of these children, with 733 (1.1%) children sustaining moderate to most severe chest injuries. One or more traumatic rib fractures were diagnosed in 202 (0.3%) children. Moderate to severe chest injuries were most frequently seen in children between 13 and 15 years of age. The same accounts for rib fractures, but the incidence of rib fractures among newborns was relatively high ($n = 11$, 5.4%) compared to the ages between 1 and 5 years (2.0%–3.5%).

**Table 1:** Age-specific incidence of traumatic chest injuries in children

Age in years	Patients admitted after a trauma, $n$ (%)	Patients admitted with any chest injury, $n$ (%)	Patients admitted with moderate to severe chest injuries, $n$ (%)	Patients admitted with rib fractures, $n$ (%)
	$n = 66,751$	$n = 1602$	$n = 733$	$n = 202$
0	6365 (9.5)	58 (3.6)	26 (3.5)	11 (5.4)
1	8121 (12.2)	89 (5.6)	29 (4.0)	5 (2.5)
2	6049 (9.1)	70 (4.4)	24 (3.3)	4 (2.0)
3	4908 (7.4)	96 (6.0)	36 (4.9)	7 (3.5)
4	4564 (6.8)	94 (5.9)	48 (6.5)	7 (3.5)
5	4409 (6.6)	85 (5.3)	33 (4.5)	4 (2.0)
6	4231 (6.3)	94 (5.9)	34 (4.6)	11 (5.4)
7	3715 (5.6)	85 (5.3)	34 (4.6)	9 (4.5)
8	3504 (5.2)	90 (5.6)	34 (4.6)	13 (6.4)
9	3123 (4.7)	77 (4.8)	30 (4.1)	10 (5.0)
10	3051 (4.6)	91 (5.7)	43 (5.9)	11 (5.4)
11	3021 (4.5)	90 (5.6)	46 (6.3)	16 (7.9)
12	3023 (4.5)	96 (6.0)	42 (5.7)	10 (5.0)
13	3080 (4.6)	141 (8.8)	73 (10.0)	18 (8.9)
14	2867 (4.3)	157 (9.8)	90 (12.3)	34 (16.8)
15	2720 (4.1)	189 (11.8)	111 (15.1)	32 (15.8)

### Baseline characteristics

A total of 733 children with chest injuries were included for further analysis. The median age was 10.9 (IQR 5.7–14.2) years and 62.6% were male (Table 2). Comorbidities were seen in 7.9% with a mild systemic disease (ASA 2) and 0.5% with moderate to severe systemic disease (ASA 3–4). Mechanisms of injury were mostly bicycle accidents (14.9%), high-energy falls (12.4%), motor vehicle accidents (11.3%), low-energy falls (10.0%), burn injuries (8.3%), and traffic accidents as pedestrians (8.2%). The percentage of bicycle accidents increased with each age category from 2.6% among 0–3-year-old children to 24.1% among the age of 12–15 years. Traffic accidents as pedestrians were seen most in the age category of 4–7 years with 11.4%, whereas the incidence was relatively low in the youngest and oldest age categories (6.1% and 7.0%, respectively). Assaults were seen in ascending numbers from the youngest to the oldest age categories, with 0.9% to 6.0%, respectively. Submersions were most prevalent among children in the age category of 4–7 years old (4.7%). In 19.4% of all children with chest injuries, the injuries were caused by other mechanisms that were known by the treating physician but classified as “other” as it did not fit to any of the specified mechanisms. In 5.7%, the mechanisms were unknown.

**Table 2:** Baseline characteristics of children with traumatic chest injuries

Variable	Total patients	Age	Age	Age	Age
		0–3 years	4–7 years	8–11 years	12–15 years
	$n = 733$	$n = 115$	$n = 149$	$n = 153$	$n = 316$
Age at trauma, median (IQR)	10.9 (5.7–14.2)	2.2 (1.1–3.2)	5.8 (4.8–7.0)	10.3 (9.2–11.3)	14.5 (13.5–15.3)
Sex, $n \left(\%\right)$					
Male	459 (62.6)	66 (57.4)	95 (63.8)	92 (60.1)	206 (65.2)
Female	273 (37.2)	48 (41.7)	54 (36.2)	61 (39.9)	110 (34.8)
Comorbidity ASA, $n \left(\%\right)$					
Normal healthy patient	573 (78.2)	79 (68.7)	128 (85.9)	127 (83.0)	239 (75.6)
Mild systemic disease	58 (7.9)	13 (11.3)	7 (4.7)	6 (3.9)	32 (10.1)
Moderate systemic disease	3 (0.4)	1 (0.9)	1 (0.7)	0 (0)	1 (0.3)
Severe systemic disease	1 (0.1)	0 (0)	0 (0)	0 (0)	1 (0.3)
Moribund patients	0 (0)	0 (0)	0 (0)	0 (0)	0 (0)
Missing, $n \left(\%\right)$	98 (13.4)	22 (19.1)	13 (8.7)	20 (13.1)	43 (13.6)
Mechanism of injury, $n \left(\%\right)$					
Motor vehicle accident	83 (11.3)	6 (5.2)	21 (14.1)	25 (16.3)	31 (9.8)
Motorcycle accident	31 (4.2)	0 (0)	3 (2.0)	2 (1.3)	26 (8.2)
Bicycle accident	109 (14.9)	3 (2.6)	13 (8.7)	17 (11.1)	76 (24.1)
Pedestrian	60 (8.2)	7 (6.1)	17 (11.4)	14 (9.2)	22 (7.0)
Low-energy fall	73 (10.0)	10 (8.7)	14 (9.4)	17 (11.1)	32 (10.1)
High-energy fall	91 (12.4)	14 (12.2)	22 (14.8)	23 (15.0)	32 (10.1)
Assault	28 (3.8)	1 (0.9)	3 (2.0)	5 (3.3)	19 (6.0)
Burn injuries	61 (8.3)	19 (16.5)	18 (12.1)	10 (6.5)	14 (4.4)
Submersion	13 (1.8)	2 (1.7)	7 (4.7)	0 (0)	4 (1.3)
Other	142 (19.4)	44 (38.3)	26 (17.4)	28 (18.3)	44 (13.9)
Missing	42 (5.7)	9 (7.8)	5 (3.4)	12 (7.8)	16 (5.1)

*IQR* interquartile range, *ASA* American Society of Anesthesiologists

### Prehospital data

Most children were transported by ambulance to the emergency department (60.8%), followed by the ambulance with deployment of a helicopter with MMT at the scene (13.6%) and own transport (12.1%) (Table 3). The youngest age category used own transport to the hospital relatively often compared to the other ages (20.0% versus 6.0–12.7%). In 25.8% of the patients, a MMT was involved at the scene of accident, which was most frequently seen among the 4–7-year-old children (32.2%) and children between 12 and 15 years (27.5%). Intubation on-scene was performed in 15.8% ($n = 116$) of the patients, and subgroup analysis showed that 33 of these children died within 30 days. Circulatory arrests were seen in 35 (4.8%) children, of whom 22 died within 30 days.

**Table 3:** Prehospital data of children with traumatic chest injuries

Variable	Total patients	Age	Age	Age	Age
		0–3 years	4–7 years	8–11 years	12–15 years
	$n = 733$	$n = 115$	$n = 149$	$n = 153$	$n = 316$
Mode of transport, $n \left(\%\right)$					
Ambulance	446 (60.8)	66 (57.4)	94 (63.1)	101 (66.0)	185 (58.5)
Own transport	89 (12.1)	23 (20.0)	9 (6.0)	17 (11.1)	40 (12.7)
Trauma helicopter	43 (5.9)	4 (3.5)	17 (11.4)	10 (6.5)	12 (3.8)
Ambulance with helicopter MMT	100 (13.6)	11 (9.6)	20 (13.4)	17 (11.1)	52 (16.5)
Ambulance with ground MMT	21 (2.9)	2 (1.7)	4 (2.7)	3 (2.0)	12 (3.8)
Other	4 (0.5)	2 (1.7)	1 (0.7)	1 (0.7)	0 (0)
Missing	30 (4.1)	7 (6.1)	4 (2.7)	4 (2.6)	15 (4.7)
Glasgow coma scale, median (IQR)	15 (15–15)	15 (15–15)	15 (15–15)	15 (15–15)	15 (14–15)
Involvement of MMT, $n \left(\%\right)$					
Yes	189 (25.8)	21 (18.3)	48 (32.2)	33 (21.6)	87 (27.5)
No	518 (70.7)	88 (76.5)	97 (65.1)	116 (75.8)	217 (68.7)
Missing	26 (3.5)	6 (5.2)	4 (2.7)	4 (2.6)	12 (3.8)
Intubation on-scene, $n \left(\%\right)$					
Yes	116 (15.8)	11 (9.6)	27 (18.1)	28 (18.3)	50 (15.8)
No	448 (61.1)	77 (67.0)	91 (61.1)	88 (57.5)	192 (60.8)
Missing	169 (23.1)	27 (23.5)	31 (20.8)	37 (24.3)	74 (23.4)
Circulatory arrest, $n \left(\%\right)$	35 (4.8)	5 (4.3)	9 (6.0)	8 (5.2)	13 (4.1)
Missing	180 (24.6)	31 (27.0)	35 (23.5)	36 (23.5)	78 (24.7)

*MMT* mobile medical team, *IQR* interquartile range

### Injury characteristics

The median ISS of the total cohort was 13 (IQR 5–22), with 42.0% being classified as polytrauma patients (Table 4). Of all children with chest injuries, 202 (27.6%) children had rib fractures and four (0.5%) children sustained a flail chest. Sternum fractures and clavicle fractures were seen in 40 (5.5%) and 60 (8.2%) children, respectively. Both the incidences of sternum fractures and clavicle fractures increased with age. Most prevalent intrathoracic injuries were lung contusions ($n = 297$, 40.5%), in contrast to the relatively rare major pneumothorax ($n = 26$, 3.5%) and major hemothorax ($n = 11$, 1.5%). Lung contusions showed the highest incidence among 4–7-year-old children (49.7%). Tracheal injuries and thoracic aorta dissections or ruptures were rare with 0.5% and 0.3%, respectively. Concomitant abdominal injuries were seen in 19.1%, and mostly in children aged 4 years or older. Concomitant spinal injuries were seen in 10.5% and occurred most frequently among the two oldest age categories.

**Table 4:** Injury characteristics of children with traumatic chest injuries

Variable	Total patients	Age	Age	Age	Age
		0–3 years	4–7 years	8–11 years	12–15 years
	$n = 733$	$n = 115$	$n = 149$	$n = 153$	$n = 316$
Rib fractures, $n \left(\%\right)$	202 (27.6)	27 (23.5)	31 (20.8)	50 (32.7)	94 (29.7)
Number of rib fractures, $n \left(\%\right)$					
One rib	73 (10.0)	10 (8.7)	11 (7.4)	16 (10.5)	36 (11.4)
Two ribs	53 (7.2)	2 (1.7)	10 (6.7)	13 (8.5)	28 (8.9)
Three or more ribs	72 (9.8)	15 (13.0)	10 (6.7)	20 (13.1)	27 (8.5)
Flail chest, $n \left(\%\right)$	4 (0.5)	0 (0)	0 (0)	1 (0.7)	3 (0.9)
Sternum fracture, $n \left(\%\right)$	40 (5.5)	2 (1.7)	2 (1.3)	9 (5.9)	27 (8.5)
Clavicle fractures, $n \left(\%\right)$	60 (8.2)	3 (2.6)	9 (6.0)	14 (9.2)	34 (10.8)
Scapula fractures, $n \left(\%\right)$	17 (2.3)	2 (1.7)	2 (1.3)	4 (2.6)	9 (2.8)
Pneumothorax, $n \left(\%\right)$	26 (3.5)	4 (3.5)	2 (1.3)	5 (3.3)	15 (4.7)
Tension pneumothorax, $n \left(\%\right)$	8 (1.1)	1 (0.9)	2 (1.3)	2 (1.3)	3 (0.9)
Hemothorax, $n \left(\%\right)$	11 (1.5)	1 (0.9)	3 (2.0)	3 (2.0)	4 (1.3)
Hemopneumothorax, $n \left(\%\right)$	1 (0.1)	0 (0)	0 (0)	1 (0.7)	0 (0)
Lung contusion, $n \left(\%\right)$	297 (40.5)	30 (26.1)	74 (49.7)	56 (36.6)	137 (43.4)
Lung contusion types, $n \left(\%\right)$					
Unilateral minor	76 (10.4)	4 (3.5)	16 (10.7)	21 (13.7)	35 (11.1)
Unilateral major	29 (4.0)	0 (0)	13 (8.7)	2 (1.3)	14 (4.4)
Bilateral minor	25 (3.4)	5 (4.3)	7 (4.7)	1 (0.7)	12 (3.8)
Bilateral major	58 (7.9)	6 (5.2)	10 (6.7)	13 (8.4)	29 (9.2)
Tracheal injury, $n \left(\%\right)$	4 (0.5)	1 (0.9)	1 (0.7)	2 (1.3)	0 (0)
Thoracic aorta dissection/rupture, $n \left(\%\right)$	2 (0.3)	1 (0.9)	0 (0)	0 (0)	1 (0.3)
Concomitant abdominal injuries, $n \left(\%\right)$	140 (19.1)	10 (8.7)	31 (20.8)	35 (22.9)	64 (20.3)
Concomitant spinal injuries, $n \left(\%\right)$	77 (10.5)	6 (5.2)	4 (2.7)	21 (13.7)	46 (14.6)
Revised trauma score, median (IQR)	7.8 (6.9–7.8)	7.8 (6.4–7.8)	7.8 (6.6–7.8)	7.8 (7.6–7.8)	7.8 (6.9–7.8)
Missing, $n \left(\%\right)$	289 (39.4)	71 (61.7)	70 (47.0)	46 (30.1)	102 (32.3)
ISS, median (IQR)	13 (5–22)	9 (4–17)	14 (6–22)	13 (5–22)	14 (6–24)
Polytrauma (ISS > 16), $n \left(\%\right)$					
Yes	308 (42.0)	33 (28.7)	67 (45.0)	62 (40.5)	146 (46.2)
No	424 (57.8)	82 (71.3)	81 (54.4)	91 (59.5)	170 (53.8)
AIS, median (IQR)					
Head	0 (0–2)	0 (0–1)	0 (0–2)	0 (0–2)	0 (0–3)
Face	0 (0–0)	0 (0–0)	0 (0–0)	0 (0–1)	0 (0–1)
Neck	0 (0–0)	0 (0–0)	0 (0–0)	0 (0–0)	0 (0–0)
Thorax	2 (2–3)	2 (2–3)	2 (2–3)	2 (2–3)	2 (2–3)
Abdomen	0 (0–0)	0 (0–0)	0 (0–0)	0 (0–0)	0 (0–0)
Spine	0 (0–0)	0 (0–0)	0 (0–0)	0 (0–0)	0 (0–0)
Upper extremity	0 (0–1)	0 (0–0)	0 (0–0)	0 (0–1)	0 (0–2)
Lower extremity	0 (0–1)	0 (0–0)	0 (0–0)	0 (0–1)	0 (0–1)
External	0 (0–0)	0 (0–0)	0 (0–0)	0 (0–0)	0 (0–0)

*IQR* interquartile range, *ISS* injury severity score, *AIS* abbreviated injury scale

### In-hospital outcomes

A total of 66.7% ($n=489$) of the children were admitted to a level 1 trauma center, and among polytrauma patients, this percentage increased to 87.3% (Table 5). Emergency interventions were 16 (2.2%) damage control thoracotomies or laparotomies and 13 (1.8%) craniotomies. The median HLOS was 3 (IQR 2–8) days, and 43.4% were admitted to the ICU with an ILOS of 3 (IQR 2–6) days. Among 110 children requiring mechanical ventilation, the duration of mechanical ventilation was 3 (IQR 1–7) days. Destinations of discharge were most frequently home (67.7%), nursing ward of another hospital (12.3%), and rehabilitation clinics (5.7%). GOS scores at discharge were good recovery in 34.9%, moderate disability in 30.8%, severe disability in 10.6%, and persistent vegetative state in 1.4% of the children. In-hospital mortality and 30-day mortality rates were 5.5% and 6.8%, respectively.

**Table 5:** In-hospital outcomes and 30-day mortality of children with traumatic chest injuries

Variable	Total patients	Age	Age	Age	Age
		0–3 years	4–7 years	8–11 years	12–15 years
	$n = 733$	$n = 115$	$n = 149$	$n = 153$	$n = 316$
Time between trauma and arrival at ED (minutes), median (IQR)	78 (51–524)	111 (53–785)	81 (52–486)	70 (49–379)	71 (50–418)
Missing, $n \left(\%\right)$	24 (3.3)	5 (4.3)	7 (4.7)	4 (2.6)	8 (2.5)
Emergency department length of stay (minutes), median (IQR)	135 (90–209)	119 (86–189)	115 (77–181)	135 (91–209)	151 (99–220)
Missing, $n \left(\%\right)$	12 (1.6)	3 (2.6)	1 (0.7)	2 (1.3)	6 (1.9)
Time between trauma and first intervention (minutes), median (IQR)	89 (56–133)	94 (52–114)	74 (54–142)	95 (77–223)	81 (55–107)
Admission to level 1 trauma center, $n \left(\%\right)$	489 (66.7)	74 (64.3)	110 (73.8)	107 (69.9)	198 (62.7)
Admission to level 1 trauma center in case of polytrauma, $n \left(\%\right)$	269 (87.3)	27 (81.8)	62 (92.5)	58 (93.5)	122 (83.6)
Emergency intervention, $n \left(\%\right)$					
Damage control thoracotomy/laparotomy	16 (2.2)	0 (0)	4 (2.7)	4 (2.6)	8 (2.5)
Craniotomy	13 (1.8)	0 (0)	2 (1.3)	3 (2.0)	8 (2.5)
Other	65 (8.8)	8 (7.0)	13 (8.7)	16 (10.5)	28 (8.9)
Missing	54 (7.4)	8 (7.0)	5 (3.4)	13 (8.5)	28 (8.9)
HLOS, median (IQR)	3 (2–8)	3 (2–6)	3 (2–8)	3 (2–8)	3 (2–8)
Missing, $n \left(\%\right)$	28 (3.8)	2 (1.7)	4 (2.7)	10 (6.6)	12 (3.8)
Admission to ICU, $n \left(\%\right)$					
Yes	318 (43.4)	43 (37.4)	78 (52.3)	65 (42.5)	132 (41.8)
No	390 (53.2)	69 (60.0)	67 (45.0)	78 (51.0)	176 (55.7)
Missing, $n \left(\%\right)$	25 (3.4)	3 (2.6)	4 (2.7)	10 (6.5)	8 (2.5)
ILOS among patients admitted to ICU, median (IQR)	3 (2–6)	3 (2–6)	3 (2–4)	3 (2–7)	3 (2–7)
Missing, $n \left(\%\right)$	1 (0.1)	1 (0.9)	0 (0)	0 (0)	0 (0)
Requiring mechanical ventilation, $n \left(\%\right)$					
Yes	110 (15.0)	17 (14.8)	22 (14.8)	23 (15.0)	48 (15.2)
No	223 (30.4)	26 (22.6)	55 (36.9)	44 (28.8)	98 (31.0)
Missing	400 (54.6)	72 (62.6)	72 (48.3)	86 (56.2)	170 (53.8)
DMV among ventilation requiring patients (days), median (IQR)	3 (1–7)	2 (1–6)	2 (1–6)	3 (1–16)	3 (1–8)
Destination after discharge, $n \left(\%\right)$					
Home	496 (67.7)	82 (71.3)	97 (65.1)	107 (69.9)	210 (66.5)
Other hospital, nursing ward	90 (12.3)	19 (16.5)	26 (17.5)	10 (6.5)	35 (11.1)
Other hospital, higher level ICU	5 (0.7)	1 (0.9)	2 (1.3)	0 (0)	2 (0.6)
Other hospital, same/lower ICU	17 (2.3)	1 (0.9)	5 (3.4)	5 (3.3)	6 (1.9)
Other hospital, foreign country	5 (0.7)	1 (0.9)	1 (0.7)	0 (0)	3 (0.9)
Rehabilitation clinic	42 (5.7)	2 (1.7)	3 (2.0)	14 (9.2)	23 (7.3)
Other	3 (0.4)	0 (0)	1 (0.7)	0 (0)	2 (0.6)
Missing	31 (4.2)	2 (1.7)	6 (4.0)	9 (5.9)	14 (4.4)
GOS score at discharge, $n \left(\%\right)$					
Good recovery	256 (34.9)	48 (41.7)	56 (37.6)	51 (33.3)	101 (32.0)
Moderate disability	226 (30.8)	30 (26.1)	42 (28.2)	46 (30.1)	108 (34.2)
Severe disability	78 (10.6)	7 (6.1)	22 (14.8)	16 (10.5)	33 (10.4)
Persistent vegetative state	10 (1.4)	1 (0.9)	1 (0.7)	4 (2.6)	4 (1.3)
Missing	123 (16.8)	23 (20.0)	20 (13.4)	27 (17.6)	53 (16.8)
In-hospital mortality, $n \left(\%\right)$	40 (5.5)	6 (5.2)	8 (5.4)	9 (5.9)	17 (5.4)
30-day mortality, $n \left(\%\right)$	50 (6.8)	7 (6.1)	10 (6.7)	11 (7.2)	22 (7.0)
Missing, $n \left(\%\right)$	167 (22.8)	26 (22.6)	37 (24.8)	29 (19.0)	75 (23.7)

*ED* emergency department, *IQR* interquartile range, *HLOS* hospital length of stay, *ICU* intensive care unit, *ILOS* intensive care unit length of stay, *DMV* duration of mechanical ventilation, *GOS* Glasgow outcome scale

## Discussion

This is a nationwide retrospective cohort study, covering a population of 17 million people with 3 million children, presenting epidemiological and clinical characteristics of pediatric trauma patients with chest injuries.

When evaluating the trend of the incidence of rib fractures among all ages, it seemed to gradually increase with age and in particular at the ages around puberty when ossification of the ribs becomes completed [11, 12]. Besides this expected trend, the incidence of rib fractures among infants was approximately twofold higher compared to the incidence at other ages under 6 years. These numbers could be a sign of child abuse, as it has been shown that rib fractures in infants are strongly suggestive for nonaccidental trauma [9, 20]. Rib fractures in infants caused by abuse have been shown to follow a certain pattern of fractures located at the bony posterior rib arcs [21]. Nevertheless, these data about the injury pattern cannot be extracted from the DTR. Other signs of nonaccidental trauma may be found in the reported mechanisms of injury. Assaults, which comprises assaults with blunt objects, sharp objects, and shooting incidents, were seen in an increasing incidence with age. But besides these numbers of injuries reportedly caused by nonaccidental trauma, the proportion of trauma mechanisms that were classified as “other” or those that were missing was particularly high among 0–3-year-old children. It could be argued that these proportions together, accounting for almost half of the trauma mechanisms in the youngest age category, also represent cases of child abuse as an extensive study in the United States reported that most victims were under 5 years of age [8].

Another particularity in the mechanisms of injury is the proportion of traumas caused by bicycle accidents, which was the most commonly seen cause of chest injuries in the Netherlands. With almost 15% of the injuries being caused by bicycle accidents, the incidence in this Dutch population was around twofold higher compared to the incidence in American pediatric trauma populations [22–24]. Submersions were seen most frequently among children aged 4–7 years, which is around the age that swimming lessons are advised in the Netherlands. It should be stated that these submersions did not directly cause chest injuries and that most chest injuries following submersions might have been caused by cardiopulmonary resuscitation.

The prehospital data presented in our cohort accentuated the severity of the injuries of pediatric chest trauma patients, as numbers of on-scene intubations, emergency interventions, and circulatory arrests were remarkably high. The proportions of emergency interventions in Dutch pediatric chest trauma patients were approximately twofold higher compared to the proportions in general pediatric trauma patients from other Western countries [25]. It could be argued that chest trauma patients require more emergency interventions than a general trauma population as especially chest injuries can quickly impair respiratory or circulatory function. These data show the impact that chest injuries have on children and therewith underline the importance of evaluating children with chest injuries cautiously.

In the total cohort, the most prevalent chest injuries were lung contusions (40.5%) and rib fractures (27.6%). The incidence of lung contusions found in this study seems in line with previous literature, as a review showed that the incidence of lung contusions in different pediatric trauma populations ranges between 43 and 73% [15]. Furthermore, it was shown that lung contusions in pediatric trauma patients may frequently exist in the absence of rib fractures or other superficial chest injuries, which was seen in this cohort as well [26, 27]. Multiple rib fractures, which have previously been shown to increase the risk of pediatric mortality with each additional rib fractured, were seen most in the age categories of 0–3 years and 8–11 years [28]. Major pneumothorax was reported in only 3.5% of the included patients, which was relatively low compared to the reported incidence of pneumothorax of 37% by other studies [13]. This contrast was most likely caused by the fact that the DTR only coded for major pneumothorax, which were described as pneumothorax with more than 50% collapse of the lung documented on chest radiography. Nonetheless, the clinical importance of documenting the much more commonly seen smaller, and no intervention requiring, pneumothorax might be considered insignificant. Major hemothorax was also infrequent in this cohort with 1.5% compared to the 13.3% hemothorax described by other literature [13]. This contrast was similarly caused by the strict coding standards from the DTR, as data on minor hemothorax were not collected and only more than 1000 cc blood loss on at least one side was considered a major hemothorax, while such volumes of blood loss are rare in the very young. Almost one in five children sustained concomitant abdominal injuries, which was previously been shown to further increase the risk of mortality [1]. Concomitant abdominal injuries and polytrauma injuries were predominantly reported among the ages above 3 years. This seems paradoxical, as it could be argued that concomitant injuries are more easily committed to the very young with little body surface. However, children under the age of 4 years in this cohort were less often injured by high-energy trauma mechanisms, such as traffic accidents, compared to children aged 4–15 years.

Two-thirds of all children with chest injuries were admitted to a level 1 trauma center, with an increased percentage among polytrauma children. However, almost one in eight pediatric polytrauma patients were not admitted to a level 1 trauma center, which may emphasize the need of adequate prehospital triage. In this cohort, children with chest injuries were admitted for a median of three days, whereas studies showed that general pediatric trauma patients and moderately to severely injured trauma patients were admitted for one and five days, respectively [5, 25]. Among the children with chest injuries, 43.4% were admitted to the ICU, with a median ILOS of three (IQR 2–6) days, which was long compared to the median ILOS of one day among pediatric trauma patients in the UK [5]. In total, the in-hospital mortality rate in our cohort was relatively high (5.5%) compared to general pediatric trauma populations (0.8–1.6%), but showing only a slightly lower mortality rate in comparison to polytrauma and moderate to severely injured pediatric trauma populations (6.6–7.2%) [5, 22, 23, 25].

In this study, a complete overview of the epidemiology, injury patterns, and outcomes of all Dutch pediatric chest trauma patients was provided. To our knowledge, no recent epidemiological study on this distinct pediatric population was performed recently. Moreover, data from the DTR covers almost all hospitals in all different regions of the Netherlands, leading to a broad heterogeneous study population. Still, this study has its limitations. First, data that were previously collected from medical records were retrospectively analyzed, resulting in sizable percentages of missing data, which may have introduced an information bias. Also, the reasons for performing specific emergency interventions and the exact causes of mortality remain unknown due to the retrospective nature of this study. Second, even though all children with chest injuries over three years were included, the incidence of certain rare injuries might still be too low for reliable analysis. Last, the incidence rates of the described injuries might have been underestimated as the DTR only registers patients who have been admitted to a hospital. Therefore, patients examined and treated at the emergency department only, without being admitted to the hospital, were not covered in this study.

In conclusion, this nationwide study shows that moderate to severe chest injuries are rare in children, especially when considering that these numbers were derived from a population of 3 million children. The most commonly seen injuries were lung contusions, followed by rib fractures. This contrasting injury pattern compared to adults underlines the importance of evaluating children with chest injuries with additional caution. This study also demonstrates inconsistencies in trauma mechanisms and injury patterns raising the suspicion of child abuse, especially in young children. The overall in-hospital mortality rate was 5.5%, and after 30 days, this percentage increased to 6.8%.

## Data Availability

The data that support the findings of this study are available from the Dutch Trauma Registration. Restrictions apply to the availability of these data, which were used under license for this study. Data can be requested at www.lnaz.nl.

## Abbreviations

AIS: Abbreviated injury scale
ASA: American Society of Anesthesiologists
DMV: Duration of mechanical ventilation
DTR: Dutch Trauma Registration
ED: Emergency department
ED-LOS: Emergency department length of stay
GCS: Glasgow coma scale
GOS: Glasgow outcome scale
HLOS: Hospital length of stay
ICU: Intensive care unit
ILOS: Intensive care unit length of stay
ISS: Injury severity score
IQR: Interquartile range
MMT: Mobile medical team
RTS: Revised trauma score
SD: Standard deviation
STROBE: Strengthening the Reporting of Observational Studies in Epidemiology
